# 2-[(*E*)-(Pyridin-2-yl­methyl­idene)amino]­thio­phene-3-carbonitrile

**DOI:** 10.1107/S1600536812043188

**Published:** 2012-11-03

**Authors:** Andréanne Bolduc, Étienne Knipping, W. G. Skene

**Affiliations:** a Department of Chemistry, Université de Montréall, CP 6128, succ. Centre-ville, Montréal, Qc, Canada

## Abstract

In the title compound, C_11_H_7_N_3_S, the thio­phene and pyridine rings are coplanar, forming a dihedral angle of 3.89 (7)°. The conformation about the C=N bond [1.2795 (18) Å] is *E*. In the crystal, translationally related mol­ecules along the *a* axis form weak π–π inter­actions [centroid–centroid distance = 3.8451 (8) Å] between the thio­phene rings.

## Related literature

For a related structure, see: Skene *et al.* (2006 ▶).
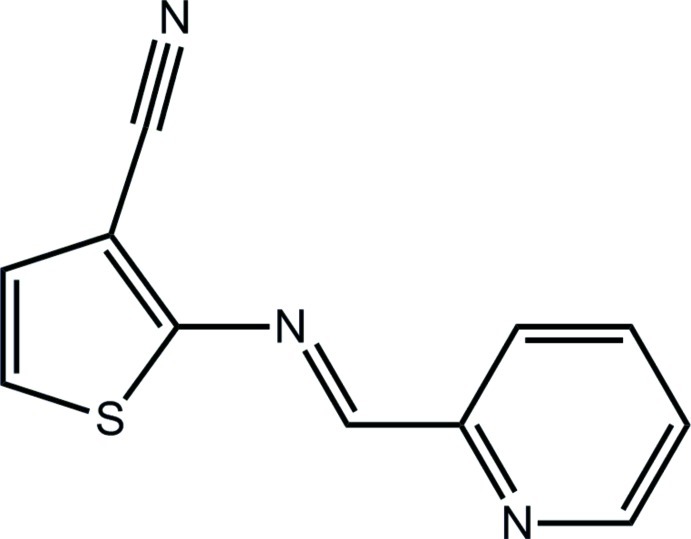


## Experimental

### 

#### Crystal data

C_11_H_7_N_3_S
*M* *_r_* = 213.26
Monoclinic, 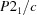

*a* = 3.8451 (1) Å
*b* = 20.8901 (4) Å
*c* = 12.2725 (2) Å
β = 94.952 (1)°
*V* = 982.10 (4) Å^3^
*Z* = 4
Cu *K*α radiation
μ = 2.64 mm^−1^
*T* = 296 K
0.18 × 0.14 × 0.13 mm

#### Data collection

Bruker SMART 6000 diffractometer
Absorption correction: multi-scan (*SADABS*; Sheldrick, 1996 ▶) *T*_min_ = 0.637, *T*_max_ = 0.710
13010 measured reflections
1940 independent reflections
1777 reflections with *I* > 2σ(*I*)
*R*_int_ = 0.037

#### Refinement

*R*[*F* ^2^ > 2σ(*F* ^2^)] = 0.035
*wR*(*F* ^2^) = 0.102
*S* = 1.08
1940 reflections
137 parameters
H-atom parameters constrained
Δρ_max_ = 0.20 e Å^−3^
Δρ_min_ = −0.24 e Å^−3^

### 

Data collection: *APEX2* (Bruker, 2009 ▶); cell refinement: *SAINT* (Bruker, 2009 ▶); data reduction: *SAINT*; program(s) used to solve structure: *SHELXS97* (Sheldrick, 2008 ▶); program(s) used to refine structure: *SHELXL97* (Sheldrick, 2008 ▶); molecular graphics: *ORTEP-3 for Windows* (Farrugia, 1997 ▶); software used to prepare material for publication: *UdMX* (Marris, 2004 ▶).

## Supplementary Material

Crystal structure: contains datablock(s) I, global. DOI: 10.1107/S1600536812043188/lx2267sup1.cif

Structure factors: contains datablock(s) I. DOI: 10.1107/S1600536812043188/lx2267Isup2.hkl

Supplementary material file. DOI: 10.1107/S1600536812043188/lx2267Isup3.cdx

Supplementary material file. DOI: 10.1107/S1600536812043188/lx2267Isup4.cml

Additional supplementary materials:  crystallographic information; 3D view; checkCIF report

## Figures and Tables

**Fig. 1.**
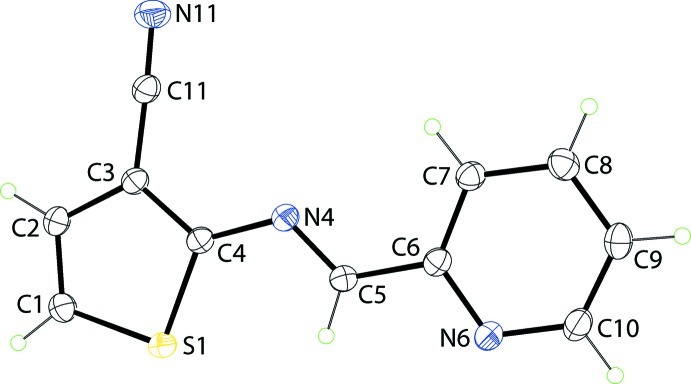
Molecular structure with the numbering scheme adopted and ellipsoids drawn at 30% probability level.

**Fig. 2.**
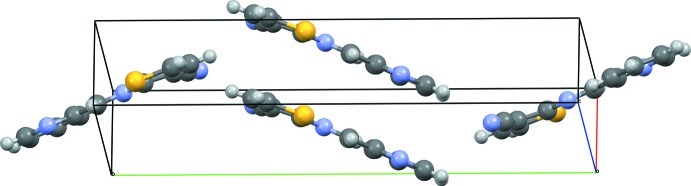
A view of the unit cell contents for (I).

## supplementary crystallographic information

### Comment

Title compound (I) was made during our ongoing research on azomethine materials. It is one of a limited number of reported crystal structures of pyridine azomethine derivatives. The structure was confirmed by the X-ray crystallography as shown in Fig. 1. The *ORTEP* diagram shows that the structure adopts the thermodynamically stable *E* isomer.

One of the major points of interest is the azomethine bond. The bond lengths for C5—C6, C5—N4 and N4—C4 are 1.463 (2), 1.2795 (18) and 1.3869 (18) Å, respectively. The bond distances are consistent with similar compounds made of thiophene units with one azomethine bond (Skene *et al.*, 2006). The bond lengths in a related molecule, *i.*e. (*E*)-diethyl 2-amino-5-(2-thienylmethyleneamino)thiophene-3,4-dicarboxylate, are 1.426 (3), 1.283 (3) and 1.381 (3) Å, respectively. The planes described by the thiophene and the pyridine moieties form a dihedral angle of 3.89 (7)° between each other.

A view of the crystal packing for (I) is illustrated in Fig. 2. Molecules stack along the *a* axis forming weak π—π interactions [3.8451 (8) Å for symmetry operation -1+*x*, *y*, *z*] formed between translationally related thiophene rings.

### Experimental

In a round bottom flask, 2-pyridinecarboxaldehyde (200 mg, 1.91 mmol) and 2-amino-3-cyanothiophene (260 mg, 2.08 mmol) were dissolved in anhydrous ethanol (25 mL). A catalytic amount of trifluoroacetic acid was added to the mixture and it was stirred at 80°C under nitrogen for 20 h. The reaction was then cooled to room temperature and the resulting product filtered to get the title compound as a yellow crystals (155.9 mg, 38%).

### Refinement

H atoms were placed in calculated positions (C—H = 0.93 Å) and included in the refinement in the riding-model approximation, with *U*_iso_(H) = 1.2*U*_eq_(C).

### Crystal data

**Table:**

C_11_H_7_N_3_S	*F*(000) = 440
*M**_r_* = 213.26	*D*_x_ = 1.442 Mg m^−^^3^
Monoclinic, *P*2_1_/*c*	Cu *K*α radiation, λ = 1.54178 Å
Hall symbol: -P 2ybc	Cell parameters from 8225 reflections
*a* = 3.8451 (1) Å	θ = 4.2–72.2°
*b* = 20.8901 (4) Å	µ = 2.64 mm^−^^1^
*c* = 12.2725 (2) Å	*T* = 296 K
β = 94.952 (1)°	Cube, yellow
*V* = 982.10 (4) Å^3^	0.18 × 0.14 × 0.13 mm
*Z* = 4	

### Data collection

**Table:**

Bruker SMART 6000 diffractometer	1940 independent reflections
Radiation source: Rotating Anode	1777 reflections with *I* > 2σ(*I*)
Montel 200 optics monochromator	*R*_int_ = 0.037
Detector resolution: 5.5 pixels mm^-1^	θ_max_ = 72.6°, θ_min_ = 4.2°
ω scans	*h* = −4→4
Absorption correction: multi-scan (*SADABS*; Sheldrick, 1996)	*k* = −25→22
*T*_min_ = 0.637, *T*_max_ = 0.710	*l* = −15→15
13010 measured reflections	

### Refinement

**Table:**

Refinement on *F*^2^	Secondary atom site location: difference Fourier map
Least-squares matrix: full	Hydrogen site location: inferred from neighbouring sites
*R*[*F*^2^ > 2σ(*F*^2^)] = 0.035	H-atom parameters constrained
*wR*(*F*^2^) = 0.102	*w* = 1/[σ^2^(*F*_o_^2^) + (0.0705*P*)^2^ + 0.1553*P*] where *P* = (*F*_o_^2^ + 2*F*_c_^2^)/3
*S* = 1.08	(Δ/σ)_max_ = 0.001
1940 reflections	Δρ_max_ = 0.20 e Å^−^^3^
137 parameters	Δρ_min_ = −0.24 e Å^−^^3^
0 restraints	Extinction correction: *SHELXL97* (Sheldrick, 2008), Fc^*^=kFc[1+0.001xFc^2^λ^3^/sin(2θ)]^-1/4^
Primary atom site location: structure-invariant direct methods	Extinction coefficient: 0.0048 (8)

### Special details

**Table:**

Experimental. X-ray crystallographic data for I were collected from a single-crystal sample, which was mounted on a loop fiber. Data were collected using a Bruker Platform diffractometer, equiped with a Bruker *SMART* 4 K Charged-Coupled Device (CCD) Area Detector using the program *APEX2* and a Nonius FR591 rotating anode equiped with a Montel 200 optics The crystal-to-detector distance was 5.0 cm, and the data collection was carried out in 512 *x* 512 pixel mode. The initial unit-cell parameters were determined by a least-squares fit of the angular setting of strong reflections, collected by a 10.0 degree scan in 33 frames over four different parts of the reciprocal space (132 frames total). One complete sphere of data was collected, to better than 0.80 Å resolution.
Geometry. All e.s.d.'s (except the e.s.d. in the dihedral angle between two l.s. planes) are estimated using the full covariance matrix. The cell e.s.d.'s are taken into account individually in the estimation of e.s.d.'s in distances, angles and torsion angles; correlations between e.s.d.'s in cell parameters are only used when they are defined by crystal symmetry. An approximate (isotropic) treatment of cell e.s.d.'s is used for estimating e.s.d.'s involving l.s. planes.
Refinement. Refinement of *F*^2^ against ALL reflections. The weighted *R*-factor *wR* and goodness of fit *S* are based on *F*^2^, conventional *R*-factors *R* are based on *F*, with *F* set to zero for negative *F*^2^. The threshold expression of *F*^2^ > σ(*F*^2^) is used only for calculating *R*-factors(gt) *etc*. and is not relevant to the choice of reflections for refinement. *R*-factors based on *F*^2^ are statistically about twice as large as those based on *F*, and *R*- factors based on ALL data will be even larger.

### Fractional atomic coordinates and isotropic or equivalent isotropic displacement parameters (Å^2^)

**Table:**

	*x*	*y*	*z*	*U*_iso_*/*U*_eq_	
S1	0.28514 (9)	0.585396 (16)	0.49120 (3)	0.03295 (17)	
N4	0.3288 (3)	0.55247 (5)	0.27296 (10)	0.0300 (3)	
N6	−0.0323 (3)	0.39582 (6)	0.24657 (11)	0.0355 (3)	
N11	0.6852 (4)	0.70387 (7)	0.16200 (12)	0.0479 (4)	
C1	0.4195 (4)	0.66118 (7)	0.52750 (12)	0.0351 (3)	
H1	0.4165	0.6777	0.5978	0.042*	
C2	0.5305 (4)	0.69438 (7)	0.44255 (12)	0.0330 (3)	
H2	0.6126	0.7362	0.4479	0.040*	
C3	0.5078 (4)	0.65807 (6)	0.34397 (12)	0.0296 (3)	
C4	0.3787 (3)	0.59711 (6)	0.35650 (12)	0.0284 (3)	
C5	0.1810 (4)	0.49926 (7)	0.29216 (12)	0.0307 (3)	
H5	0.1096	0.4923	0.3617	0.037*	
C6	0.1192 (4)	0.44890 (7)	0.20990 (12)	0.0299 (3)	
C7	0.2076 (4)	0.45613 (7)	0.10312 (12)	0.0331 (3)	
H7	0.3145	0.4934	0.0816	0.040*	
C8	0.1335 (4)	0.40665 (7)	0.02923 (14)	0.0376 (4)	
H8	0.1885	0.4101	−0.0429	0.045*	
C9	−0.0248 (4)	0.35197 (7)	0.06573 (14)	0.0375 (4)	
H9	−0.0810	0.3182	0.0181	0.045*	
C10	−0.0979 (4)	0.34840 (7)	0.17395 (14)	0.0387 (4)	
H10	−0.1983	0.3110	0.1978	0.046*	
C11	0.6052 (4)	0.68224 (7)	0.24209 (13)	0.0343 (3)	

### Atomic displacement parameters (Å^2^)

**Table:**

	*U*^11^	*U*^22^	*U*^33^	*U*^12^	*U*^13^	*U*^23^
S1	0.0387 (2)	0.0311 (2)	0.0296 (2)	−0.00353 (13)	0.00597 (16)	0.00229 (12)
N4	0.0314 (6)	0.0272 (6)	0.0315 (6)	−0.0004 (4)	0.0028 (5)	0.0014 (5)
N6	0.0401 (7)	0.0303 (6)	0.0359 (7)	−0.0054 (5)	0.0016 (5)	0.0021 (5)
N11	0.0656 (10)	0.0385 (8)	0.0412 (8)	−0.0017 (7)	0.0133 (7)	0.0070 (6)
C1	0.0394 (8)	0.0340 (8)	0.0323 (8)	−0.0001 (6)	0.0043 (6)	−0.0036 (6)
C2	0.0349 (7)	0.0276 (7)	0.0364 (8)	−0.0010 (5)	0.0025 (6)	−0.0020 (5)
C3	0.0306 (7)	0.0262 (7)	0.0319 (7)	0.0005 (5)	0.0026 (5)	0.0021 (5)
C4	0.0272 (7)	0.0271 (7)	0.0309 (7)	0.0011 (5)	0.0022 (5)	0.0024 (5)
C5	0.0334 (7)	0.0291 (7)	0.0295 (7)	−0.0016 (5)	0.0024 (5)	0.0027 (5)
C6	0.0283 (6)	0.0273 (7)	0.0337 (7)	0.0009 (5)	−0.0001 (5)	0.0025 (5)
C7	0.0353 (7)	0.0286 (7)	0.0357 (7)	0.0010 (5)	0.0048 (6)	0.0015 (6)
C8	0.0414 (8)	0.0360 (8)	0.0357 (8)	0.0056 (6)	0.0053 (6)	−0.0019 (6)
C9	0.0370 (8)	0.0303 (8)	0.0444 (9)	0.0033 (6)	−0.0013 (6)	−0.0068 (6)
C10	0.0424 (8)	0.0279 (7)	0.0452 (9)	−0.0053 (6)	−0.0004 (7)	0.0005 (6)
C11	0.0402 (8)	0.0256 (7)	0.0374 (8)	−0.0011 (6)	0.0048 (6)	0.0009 (6)

### Geometric parameters (Å, º)

**Table:**

S1—C1	1.7122 (15)	C3—C11	1.428 (2)
S1—C4	1.7392 (15)	C5—C6	1.463 (2)
N4—C5	1.2795 (18)	C5—H5	0.9300
N4—C4	1.3869 (18)	C6—C7	1.390 (2)
N6—C10	1.342 (2)	C7—C8	1.388 (2)
N6—C6	1.3478 (19)	C7—H7	0.9300
N11—C11	1.147 (2)	C8—C9	1.387 (2)
C1—C2	1.352 (2)	C8—H8	0.9300
C1—H1	0.9300	C9—C10	1.383 (2)
C2—C3	1.424 (2)	C9—H9	0.9300
C2—H2	0.9300	C10—H10	0.9300
C3—C4	1.3802 (19)		
			
C1—S1—C4	91.99 (7)	C6—C5—H5	118.5
C5—N4—C4	118.89 (12)	N6—C6—C7	123.51 (13)
C10—N6—C6	116.58 (13)	N6—C6—C5	114.22 (13)
C2—C1—S1	112.46 (11)	C7—C6—C5	122.26 (13)
C2—C1—H1	123.8	C8—C7—C6	118.79 (14)
S1—C1—H1	123.8	C8—C7—H7	120.6
C1—C2—C3	112.43 (13)	C6—C7—H7	120.6
C1—C2—H2	123.8	C9—C8—C7	118.26 (15)
C3—C2—H2	123.8	C9—C8—H8	120.9
C4—C3—C2	113.14 (13)	C7—C8—H8	120.9
C4—C3—C11	123.22 (13)	C10—C9—C8	119.06 (14)
C2—C3—C11	123.64 (13)	C10—C9—H9	120.5
C3—C4—N4	124.48 (13)	C8—C9—H9	120.5
C3—C4—S1	109.97 (11)	N6—C10—C9	123.78 (14)
N4—C4—S1	125.54 (10)	N6—C10—H10	118.1
N4—C5—C6	123.08 (13)	C9—C10—H10	118.1
N4—C5—H5	118.5	N11—C11—C3	177.44 (16)
			
C4—S1—C1—C2	0.03 (12)	C4—N4—C5—C6	−179.46 (12)
S1—C1—C2—C3	0.06 (17)	C10—N6—C6—C7	−0.1 (2)
C1—C2—C3—C4	−0.15 (19)	C10—N6—C6—C5	179.36 (13)
C1—C2—C3—C11	179.33 (13)	N4—C5—C6—N6	178.52 (13)
C2—C3—C4—N4	179.51 (12)	N4—C5—C6—C7	−2.0 (2)
C11—C3—C4—N4	0.0 (2)	N6—C6—C7—C8	0.8 (2)
C2—C3—C4—S1	0.17 (16)	C5—C6—C7—C8	−178.60 (13)
C11—C3—C4—S1	−179.31 (11)	C6—C7—C8—C9	−0.3 (2)
C5—N4—C4—C3	−174.38 (13)	C7—C8—C9—C10	−0.9 (2)
C5—N4—C4—S1	4.86 (19)	C6—N6—C10—C9	−1.2 (2)
C1—S1—C4—C3	−0.12 (11)	C8—C9—C10—N6	1.7 (2)
C1—S1—C4—N4	−179.45 (12)		
